# Lack of evidence for qualitative treatment by disease severity interactions in clinical studies of severe sepsis

**DOI:** 10.1186/cc3795

**Published:** 2005-09-22

**Authors:** William L Macias, David R Nelson, Mark Williams, Rekha Garg, Jonathan Janes, Andreas Sashegyi

**Affiliations:** 1 Senior Medical Director, Lilly Research Laboratories, Indianapolis, Indiana, USA; 2 Associate Senior Statistician, Lilly Research Laboratories, Indianapolis, Indiana, USA; 3 Associate Medical Director, Lilly Research Laboratories, Indianapolis, Indiana, USA; 4 Medical Fellow, Lilly Research Laboratories, Indianapolis, Indiana, USA; 5 Senior Statistician, Lilly Research Laboratories, Indianapolis, Indiana, USA

## Abstract

**Introduction:**

The design of clinical trials of interventions aimed at reducing mortality in patients with severe sepsis assumes that the relative treatment effect of the intervention is independent of the patients' risk for death. We reviewed published data from phase III clinical studies of severe sepsis to determine whether a relationship exists between risk for death and the relative benefit of the investigational agent. Such an interaction might warrant a change in the assumptions that underlie current trial designs.

**Methods:**

We conducted a systematic review of published phase III, randomized, placebo-controlled trials in adult patients with sepsis, severe sepsis, or septic shock up to November 2004. All studies enrolled patients with known or suspected infection, evidence of a systemic response to the infection, and one or more organ dysfunctions resulting from the systemic response.

**Results:**

Twenty-two publications, investigating 17 molecular entities, fulfilled criteria for phase III or equivalent studies aimed at reducing mortality in adult patients with severe sepsis or septic shock. Three studies achieved the prospectively defined primary end-point of a statistically significant reduction in 28-day all-cause mortality. The control group mortality rates for these studies were 31%, 43% and 61%, indicating that the beneficial effects of adjunct therapies could be demonstrated over a wide range of illness severity. Analysis of subgroup data from failed studies provided no evidence that the efficacy of the therapeutics being investigated varied by baseline placebo mortality rates. Among all studies, interventions with anticoagulant activity or anti-inflammatory activity did not appear to be harmful in patients with evidence of less coagulopathy or less inflammation.

**Conclusion:**

Our review of published clinical data does not support the hypothesis that mortality risk of the population studied alters the relative treatment effect associated with anti-inflammatory or other agents used to treat severe sepsis. Clinical studies in severe sepsis should continue to enroll patients over a wide range of disease severity, as long as patients enrolled have evidence of sepsis-induced organ dysfunction(s), patients are at an appreciable risk for death (e.g. as evidenced by admission to an intensive care unit), and the potential for benefit outweighs the potential for harm.

## Introduction

The development of agents aimed at reducing mortality from severe sepsis has been predicated on the hypothesis that death results from sepsis-induced organ dysfunction, the latter being the consequence of an excessive or uncontrolled host response to the infection [1-3]. Fundamental to this hypothesis is the assumption that the host response, at least to some extent, is no longer beneficial once organ dysfunction ensues and that modulation of this response will reduce the severity of organ dysfunction or prevent additional dysfunctions [4]. Therefore, current trial designs allow the enrollment of a heterogeneous population of patients with varying numbers of organ dysfunctions, severity of illness scores, and predicted risk for death [5].

Recent publications [6-8] have challenged this hypothesis, suggesting that the host response may only be detrimental in patients with the most severe degrees of organ dysfunction and highest risk for death. As a potential result, biologic response modifiers, specifically those with anti-inflammatory effects, may only be beneficial in the most severely ill patients and could potentially be ineffective or detrimental in patients with severe sepsis and less severe organ dysfunctions [7]. The idea that biologic response modifiers might exhibit qualitative treatment effects in severe sepsis (i.e. produce beneficial effects in the most severely ill and detrimental effects in the least severely ill) is based primarily on preclinical animal studies and on *post hoc *analyses of successful and failed clinical trials in patients with severe sepsis [7]. However, a recent meta-analysis of steroid treatment in patients with sepsis and septic shock [9] failed to identify a relationship between increasing treatment benefit associated with steroid therapy and increasing control group mortality.

We therefore undertook a systematic review of all published phase III, randomized, controlled clinical trials in adult patients with severe sepsis or septic shock to determine whether there were data supporting the hypothesis that biologic modifiers might be associated with qualitative treatment effects dependent on disease severity (as assessed by control mortality rates). Understanding whether data from prior clinical trials suggest that these agents might produce differential effects on survival depending on a patient's severity of illness is important in designing future trials of newer agents in severe sepsis. We report the lack of any such data and discuss the advantages and disadvantages of current trial designs in severe sepsis.

## Materials and methods

Publications of randomized, placebo-controlled phase III or phase III equivalent studies that tested the effects of specific pharmaceutical interventions aimed at improving survival from severe sepsis were identified by a search of the PubMed database. The following search terms were used, each with restrictions for human studies and randomized controlled trials: sepsis and mortality, and severe sepsis and mortality. An additional check of the PubMed database was conducted using the search terms sepsis or severe sepsis, with restrictions for human studies and meta-analysis. Reference lists from these latter publications were cross-checked against the original search results to identify any additional reports. The PubMed database was searched multiple times throughout the preparation of this manuscript. The final search was conducted on 29 November 2004.

Studies were included in this analysis if they met the following criteria: randomized, double blind, placebo controlled clinical trial; enrollment of adult patients who met the diagnosis of severe sepsis or septic shock; assessment of 28- to 30-day all-cause mortality as the primary outcome; and adequate power (≥ 80%) to detect statistically significant improvements in the primary outcome at the two-sided alpha of 0.05. Studies that compared more than one active therapy arm with placebo were required to include an intent to adjust statistically for two or more comparisons (e.g. Bonferroni procedure) [10]. Likewise, appropriate correction for repeated comparisons at planned interim analyses (e.g. O'Brien–Flemming) was also required to have been prospectively defined if there was a possibility of stopping the study early because of efficacy [10]. The inclusion of these statistical requirements was to ensure appropriate rigor in the conduct of the study. Phase III or phase III equivalent studies were considered large enough to allow statistical interpretation of the overall population and, more importantly, of reported subgroups.

Severe sepsis was defined in all studies as follows: the presence of known or suspected infection; evidence of a systemic response to infection (e.g. fever, hypothermia, tachypnea, tachycardia, leukocytosis or leukopenia); and one or more organ dysfunctions resulting directly from the systemic response to infection (most commonly cardiovascular, respiratory, renal, hematologic or metabolic acidosis). Septic shock was defined as the presence of either hypotension (absolute or relative) or the need for vasopressor support to maintain adequate perfusion and evidence of end-organ hypoperfusion.

The primary end-point of 28-day all-cause mortality was extracted from all studies with no adjustment for imbalance in baseline characteristics between patient treatment groups. Quantitative assessments of outcome at 28 days for subgroups defined by baseline measures of disease severity were also extracted. These subpopulations included groups defined by Simplified Acute Physiology Score [11], Acute Physiology and Chronic Health Evaluation (APACHE) II [12], presence or absence of shock, presence or absence of hypotension, presence or absence of acute respiratory distress syndrome, IL-6 concentration, cardiovascular Sepsis-related Organ Failure Assessment score [13], and presence of single or multiple organ failures. Qualitative assessment of any interaction beween treatment and disease severity was extracted from the results or discussion section of the report.

Data pertaining to the safety of the intervention was also extracted. In particular, the incidence of any post-treatment infectious complications was specifically sought.

### Statistical methods

Mortality rates were extracted from publications. Some reports included the total number of patients within severity classes but did not include per treatment sample sizes within severity groups. In these instances, calculations of placebo and treatment sample sizes per groups assumed that patients were evenly divided between treatment groups. The information extracted was used in a logistic regression to determine whether there was a significant interaction between treatment and severity after adjusting for overall treatment and severity effects. One severity classification was selected per study. If multiple severity classes were reported, priority was attributed in the following order: predicted risk for death; APACHE II; shock versus no shock; and remaining available severity measure. Analyses were performed using SAS version 8.02 software (SAS Institute Inc, Cary, NC, USA).

## Results

Using the restrictions listed above, 535 and 158 publications were identified for sepsis + mortality and severe sepsis + mortality, respectively. These publications were grouped as potential phase III studies of biologic response modifiers in severe sepsis (*n *= 43), non-phase III studies of biologic response modifiers in severe sepsis (*n *= 158), antibiotic studies in severe sepsis (*n *= 76), nonantibiotic, nonbiologic response modifier studies in severe sepsis (*n* = 41), and unrelated studies (*n *= 335). A total of 110 unique reports were identified using the search terms sepsis or severe sepsis and restricted to meta-analyses of human studies, of which nine were specific to severe sepsis. From the initial publication list and review of the references from identified meta-analyses, 22 reports, investigating 17 molecular entities, fulfilled criteria for phase III or equivalent studies aimed at reducing mortality in adult patients with severe sepsis or septic shock (Table 1). A number of additional studies were identified but were not included because they were not considered phase III studies (for example [14-18]), because they lacked statistical adjustment for multiple comparisons (e.g. [19,20]), or because the 28- to 30-day mortality data were not provided (e.g. [21-23]). Supplemental publications from some studies were reviewed to extract subgroup mortality data [6,24]. Studies were conducted between January 1987 and July 2003 (Table 1). Table 2 lists the overall and subgroup results for all identified studies.

**Figure 1:**
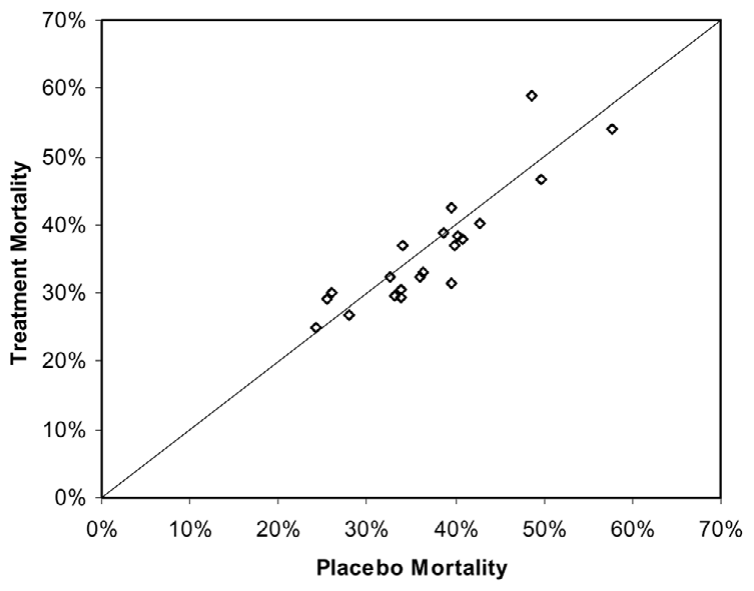
Distribution of treatment and placebo mortalities for unsuccessful sepsis trials.

Three studies met the prospectively defined primary end-point of a statistically significant reduction in 28-day all-cause mortality, namely those by Ziegler and coworkers in 1991 [25], Bernard and colleagues in 2001 [26] and Annane and coworkers in 2002 [27]. The control group mortality rates for these three studies were 43%, 31% and 61%, respectively, indicating that the beneficial effects of adjunct therapies could be demonstrated over a wide range of illness severity. Figure 1 shows the results of all trials that failed to meet their primary end-point as prospectively specified in the methods section of each report. The distribution of outcome results for placebo and active treatment groups reside along the line of unity over a placebo mortality range between 20% and 60%. These data do not suggest that a possible explanation for the lack of demonstrated efficacy in these studies resulted from either enrollment of less severe or more severely ill patients (as assessed by the observed placebo mortality rates).

**Figure 2:**
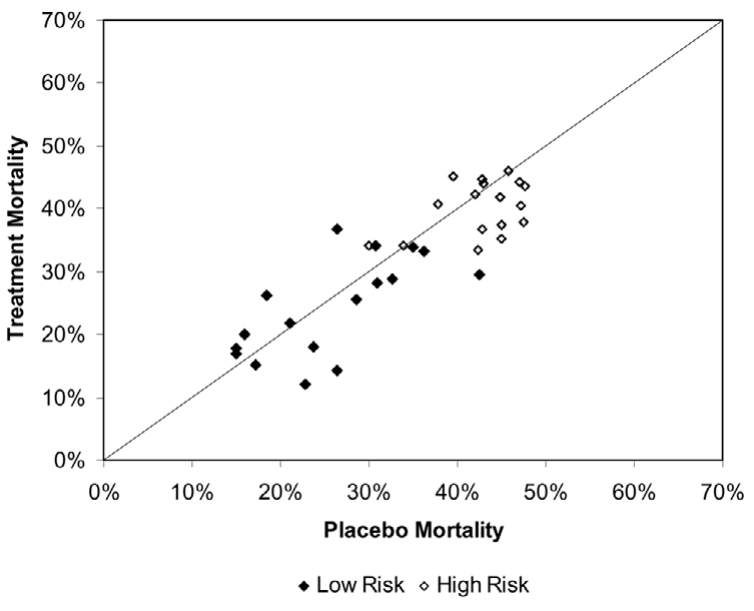
Distribution of treatment and placebo mortalities for sepsis trials by low and high risk patients.

Figure 2 shows the subgroup results, as defined by measures of disease severity, from the failed trials referred to above. Again, there is no evidence that the potential efficacy of the therapeutics within these subgroups varied by baseline placebo mortality rates. Logistic regression indicates that although patient severity is related to mortality (*P *< 0.0001), neither treatment (*P *= 0.32) nor an interaction between treatment and severity of illness (*P *= 0.70) was significantly related to mortality. For failed studies reporting survival data for subgroups defined by baseline measures of disease severity, four demonstrated lower mortality in the active treatment arm in subgroups with lower severity of illness. These were the studies by Opal and coworkers in 2004 [28], Abraham and colleagues in 2003 [29], Greenman and coworkers in 1991 [30] and Abraham and colleagues in 1995 [31] (Table 2). In two studies better outcomes were observed in higher risk subgroups whereas higher mortality was observed in the active treatment arms compared with placebo for some of the 'lower risk' subgroups: Fisher and coworkers (1994) [32], Knaus and Harrell (1996) [6], and Abraham and colleagues (2001) [33]. In the first IL-1 receptor antagonist (IL-1ra) study, lower mortality in the IL-1ra treatment group compared with placebo was observed for subgroups with a predicted risk for death of 24% or greater, regardless of dose [6]. However, in the follow-up study that sought to validate this observation [34] the opposite trend was observed.

In the study of drotrecogin alfa (activated), better outcomes were observed in higher severity subgroups defined by APACHE II scoring and in lower severity subgroups defined by biologic markers of disease severity (i.e. IL-6 level and prothrombin time) [24]. For patients enrolled in the HA-1A study [25] lower mortality was observed in the active treatment arm than in the placebo group. The observed treatment effect was evident in patients with and without shock and with APACHE II scores above and below 25. The study by Annane and colleagues [27] did not report outcomes for subgroups defined by disease severity.

Among all studies, interventions with anticoagulant activity or anti-inflammatory activity did not appear to be harmful in patients with evidence of less coagulopathy (as assessed by coagulation tests) or less inflammation (as assessed by IL-6 levels). Three studies of medications with anticoagulant properties were reported [26,29,35]. For both studies in which outcome was reported for subgroups defined by baseline prothrombin times [26,29], the observed relative reduction in the risk for death approached 50% for patients with normal coagulation status (international normalized ratio <1.2 or prothrombin time <14.5 s). Three studies reported outcomes by baseline IL-6 levels [24,26,36,37]. In the study of drotrecogin alfa (activated) [24] large absolute and relative reductions in mortality were observed in patients with the lowest IL-6 levels, whereas IL-6 levels did not appear to influence the outcome of therapy with BAYx1351 or afelimomab [36,37].

### Safety assessments

Table 3 lists pertinent findings of the safety assessments conducted in each study. Current methodology for reporting and analyzing adverse events captured a number of safety concerns associated with multiple therapies. An increase in the incidence of bleeding complications was noted for all medications with anticoagulant properties. Complications related to allergic reactions were noted for some murine-based proteins. An increase in the incidence of serious cardiac adverse events was noted in a study of a nitric oxide synthase inhibitor. None of the studies listed detected an increase in the incidence of infectious complications related to the administration of medications with either anti-inflammatory and/or anticoagulant properties.

## Discussion

Recent publications and editorials have suggested that one possible explanation for the discordance between the preclinical efficacy and subsequent clinical failures of many therapeutics investigated in severe sepsis is that these therapies might be expected to reduce mortality only in the most severely ill patients or those patients at highest risk for death [6-8]. Implicit in this explanation is that these therapies must also produce a harmful effect in the 'less severe population' because a benefit was not observed in the overall population. We investigated whether evidence for an interaction between treatment and severity exists within published clinical data from phase III studies of these agents in severe sepsis.

Twenty-two publications investigating 17 different pharmacotherapeutic agents targeting the host response to infection were identified. Only phase III studies were included to reduce potential noise related to small sample size and multiple dosing regimens. Three studies met their prospectively defined end-point. The control mortality rates range between 31% and 61%, indicating that the beneficial effects of adjunct therapies could be demonstrated over a wide range of illness severity. For failed trials, lower control mortality rates did not appear to be an explanation for possible failures (Fig. 1). Furthermore, exploration of the reported subgroups for these studies also failed to demonstrate any possible interaction between treatment and disease severity that could have contributed to the lack of observed efficacy (Fig. 2).

The first phase III study of IL-1ra has frequently been cited as evidence for the existence of a differential effect of treatment based on disease severity [7,32]. In that study a nonsignificant reduction in mortality was observed in the overall population. For patients with a predicted risk of death of 24% or greater, mortality was significantly lower in the active treatment arm than in the placebo group [6]. Higher mortality was observed in the active treatment arm for patients with a predicted risk of death below 24%. However, in the confirmatory study [34] the exact opposite was observed. A beneficial effect of treatment was observed in patients with a predicted risk for death below 24% (18% mortality for IL-1ra treated patients versus 24% mortality for placebo treated patients), whereas no difference was observed in patients with a 24% or greater predicted risk for death (42% in both treatment arms).

Additionally, interventions with anticoagulant activity or anti-inflammatory activity did not appear to be harmful in patients with evidence of less coagulopathy (as assessed by coagulation tests) or less inflammation (as assessed by IL-6 levels). Almost all investigated therapeutics had some reported anti-inflammatory effects. However, in the three studies that reported outcomes by baseline IL-6 levels [26,36,37] the observed treatment effect was either greater for patients with the lowest IL-6 levels or was unrelated to IL-6 level. In the study of drotrecogin alfa (activated) [24] large absolute and relative reductions in mortality were observed in patients with the lowest IL-6 levels. For studies of interventions with anticoagulant properties, outcomes appeared more favorable for the active treatment arms in patients with less coagulopathy at the time of study entry [24,29]. These observations suggest, with the limitations of subgroup analyses applied, that interventions with anti-inflammatory or anticoagulant properties are probably not harmful in patients with less inflammation or less coagulopathy.

Review of the safety data from each of the published studies indicates that the current reporting system for adverse events appears to be adequate in capturing potential toxicities associated with these therapies. An increased risk for bleeding complications was noted for antithrombin III, tissue factor pathway inhibitor, and drotrecogin alfa (activated), which is consistent with their anticoagulant properties. Multiple studies investigating murine-based antibodies documented allergic and anaphylactic reactions associated with therapy. Antibody formation was also documented. The nitric oxide synthase inhibitor study, in which statistically higher mortality was observed in the active treatment arm, reported a higher incidence of cardiovascular related deaths in the active treatment arm. No study reported adverse events that might be considered related to inhibition of the host response to infection. However, in the study conducted by Bone and coworkers [22] of high dose methylprednisolone (primary end-point: 14-day mortality), mortality attributed to the secondary infections was significantly increased in the methylprednisolone group than in the placebo group. Taken together, these data indicate that, as designed, clinical trial databases capture potential drug-related events and complications associated with investigational therapies.

Interactions between treatment and disease severity can be generally classified into two major categories: quantitative interactions, in which the relative benefit of a drug may be less in less severe disease; and qualitative interactions, in which the drug is truly beneficial at one level of severity and truly detrimental at another. For quantitative interactions a more favorable benefit:risk ratio might exist for patients with more severe disease, assuming that the absolute benefit of therapy is greater for more severely ill patients and that the absolute risk of therapy is uniformly distributed across disease severities. This type of interaction is not uncommon, and physicians and other health care providers commonly weigh the benefits and risks associated with all medicines before administration. Drotrecogin alfa (activated) may exhibit a quantitative interaction particularly from a risk:benefit perspective, because absolute reductions in mortality are larger for populations at higher risk for death whereas the bleeding complications of therapy appear to be independent of disease severity. As a consequence, many regulatory agencies limited the use of drotrecogin alfa (activated) to patients with severe sepsis at higher risk of death [38,39].

Qualitative interactions, on the other hand, are probably rare [40]. This type of interaction suggests that the biologic effect of the drug (e.g. an anti-inflammatory effect) is beneficial in high disease severity and that the same biologic effect is detrimental in low disease severity. A systematic review of the available published literature does not support the hypothesis that such a qualitative interaction between treatment and severity exists for compounds that target the host response to infection.

Understanding whether data from prior clinical trials suggest that these agents might produce differential effects on survival depending on a patient's severity of illness is important in designing future trials of newer agents in severe sepsis. If present, such a qualitative interaction could warrant a change in the design of phase III trials that currently enroll patients with severe sepsis over a wide range of disease severity (e.g. one to five organ dysfunctions) and predicted risk for death (between 20% and 60% mortality at 28 days from the start of treatment). Additionally, current recommendations for the treatment of severe sepsis assume, to some degree, the absence of interactions between treatment and disease severity. For example, intensive insulin therapy was demonstrated to reduce mortality in a population of nonseptic patients at very low risk for death [41]. Intensive insulin therapy was provided a grade B recommendation for the treatment of patients with severe sepsis [42] – a population of patients at much higher risk for death. Low-dose steroid therapy reduced mortality in a population of patients with severe sepsis and refractory septic shock (hypotension despite vasopressor administration; placebo mortality 63%) [27]. Low-dose steroid therapy was given a grade C recommendation [42] for the treatment of patients with severe sepsis requiring vasopressor therapy but not necessarily with persistent hypotension. In clinical studies, patients who require only vasopressor support have mortality rates ranging between 26% and 43%, depending on vasopressor dose [24,29,32,35].

The lack of evidence for a qualitative interaction between treatment and severity does not preclude that such an interaction may indeed exist. The recently completed study of drotrecogin alfa (activated) in patients with severe sepsis at lower risk of death (ADDRESS) [43] was stopped because of futility, indicating an inability to demonstrate efficacy in low risk patients. The futility of the study might have been driven by an adverse outcome in surgical patients with a single organ dysfunction, but the unfavorable outcome in surgical patients with a single organ dysfunction might not have been driven by lower severity *per se*, because a similar outcome was not observed in medical patients with a single organ dysfunction. Other potential confounders might include difficulty in making a diagnosis of severe sepsis in postoperative patients with a single organ dysfunction, a delay in treatment of these patients because of the requirement to delay therapy for 12 hours after surgery, and a higher risk for bleeding complications. This adverse finding prompted revision of the product label for drotrecogin alfa (activated) [38,39].

The observations from the ADDRESS study [43] underscore the need for studies to enroll a heterogeneous population of patients to investigate the safety and efficacy of biologic response modifiers. In the absence of such investigation, physicians will be forced to extrapolate data across populations of critically ill patients, as has been done with insulin and steroid therapy [42]. Consequently, sponsors and principal investigators should consider increasing the sample size for phase III studies beyond that necessary to detect the treatment effect in the overall population in order to allow more robust assessment of treatment effects across clinically relevant subgroups. The use of power calculations conditional on a statistically significant treatment effect being observed in the overall trial might be useful. We would also recommend that the need to assess treatment effects across subgroups be considered when designing the efficacy stopping rules for interim analyses of large phase III studies.

There are multiple limitations to the above analyses. As with any analysis based on literature review, there is always a concern regarding publication bias. Furthermore, analyses based on *post hoc *subgroups is also biased because those subgroups reported in publications of negative trials may represent those in which a beneficial treatment effect was observed in at least one stratum, leaving an unfavorable effect in the complementary strata. The study of HA-1A was included in this analysis despite concerns over the statistical rigor of the study because the intent of the present analysis was not to demonstrate that any therapy was or was not effective but to determine whether evidence supporting an interaction between treatment and disease severity exists amongst published clinical data. A follow-up study of HA-1A in a similar population of patients with severe sepsis was stopped because of futility [21]. The follow-up study was not included in this analysis because it reported only 14-day all-cause mortality as the primary end-point.

## Conclusion

A review of published clinical data does not support the hypothesis that there is a qualitative interaction between treatment and severity associated with anti-inflammatory or other agents used to treat severe sepsis. Clinical studies in severe sepsis should continue to enroll patients over a wide range of disease severity and risk for death, as long as patients enrolled have evidence of sepsis-induced organ dysfunction(s), patients are at an appreciable risk for death (e.g. as evidenced by admission to an intensive care unit), and the potential for benefit outweighs any potential for harm.

## Key messages

• 	Severe sepsis trials assume that the relative treatment effect of an intervention is independent of the patients' risk for death.

• 	Three studies met the prospectively defined primary end-point of a statistically significant reduction in 28-day all-cause mortality.

• 	The lack of demonstrated efficacy in the negative studies is not due to enrollment of less severe or more severely ill patients.

• 	Interventions with anticoagulant activity or anti-inflammatory activity did not appear to be harmful in patients with evidence of less coagulopathy or inflammation.

• 	Clinical studies in severe sepsis should continue to enroll patients over a wide range of disease severity.

## Abbreviations

APACHE = Acute Physiology and Chronic Health Evaluation; IL = interleukin; IL-1ra = IL-1 receptor antagonist.

## Competing interests

All authors are employees and shareholders of Eli Lilly and Company, who hold a patent in activated protein C.

## Authors' contributions

WM conceived the design and methods of this study and drafted the manuscript. DN performed all statistical analyses and participated in manuscript preparation. MW helped perform literature review and drafted/edited the manuscript. RG helped perform literature review and drafting of manuscript. JJ helped perform literature review and drafting of manuscript. AS assisted DN with statistical analyses and drafting of manuscript. All authors read and approved the final manuscript.

## Figures and Tables

**Table 1:** Characteristics of included randomized placebo-controlled clinical studies

Study	Molecular class	Design	Primary outcome measure
Opal *et al*. (2004) [28]	Platelet activating factor hydrolase	2 Parallel groups	28-Day all-cause mortality
Abraham *et al*. (2003) [29]	Tissue factor pathway inhibitor	2 Parallel Groups	28-Day all-cause mortality
Annane *et al*. (2002) [27]	'Low-dose' hydrocortisone plus fludrocortisone	2 Parallel groups Subset by 'responder' to cortisyn stimulation test	28-Day all-cause mortality
Warren *et al*. (2001) [35]	Antithrombin III	2 Parallel groups	28-Day all-cause mortality
Bernard *et al*. (1997) [44]	Nonsteroidal anti-inflammatory drug (ibuprofen)	2 Parallel groups	28-Day all-cause mortality
Fisher *et al*. (1994) [32]	IL-1ra	3 Parallel groups (2 active treatment arms)	28-Day all-cause mortality
Opal *et al*. (1997) [34]	IL-1ra	2 Parallel groups	28-Day all-cause mortality
Greenman *et al*. (1991) [30]	Antiendotoxin antibody (E5)	2 Parallel groups Subset by Gram-negative infection	28-Day all-cause mortality
Bone *et al*. (1995) [22]	Antiendotoxin antibody (E5)	2 Parallel groups	28-Day all-cause mortality
Angus *et al*. (2000) [45]	Antiendotoxin antibody (E5)	2 Parallel groups	28-Day all-cause mortality
Abraham *et al*. (2001) [33]	p55 TNF receptor fusion protein (lenercept)	2 Parallel groups	28-Day all-cause mortality
Reinhart *et al*. (2001) [46]	Anti-TNF antibody (MAK195F)	2 Parallel groups IL-6 > 1,000 pg/ml	28-Day all-cause mortality
Cohen and Carlet (1996) [47]	Anti-TNF antibody (BAYx1351)	3 Parallel groups	28-Day all-cause mortality
Abraham *et al*. (1995) [31]	Anti-TNF antibody (BAYx1351)	3 Parallel groups	28-Day all-cause mortality
Abraham *et al*. (1998) [36]	Anti-TNF antibody (BAYx1351)	2 Parallel groups	28-Day all-cause mortality
Bernard *et al*. (2001) [26]	Activated protein C	2 Parallel groups	28-Day all-cause mortality
Dhainaut *et al*. (1998) [48]	Platelet activating factor receptor antagonist	2 Parallel groups	28-Day all-cause mortality
Albertson *et al*. (2003) [49]	Anti-Enterobacteriaceae common antigen antibody	2 Parallel groups Subset by Enterobacteriaceae infection	28-Day all-cause mortality
Lopez *et al*. (2004) [50]	Nitric oxide synthase inhibitor	2 Parallel groups	28-Day all-cause mortality
Ziegler *et al*. (1991) [25]	Antiendotoxin antibody (HA-1A)	2 Parallel groups Subset by Gram-negative bacteremia	28-Day all-cause mortality
Panacek *et al*. (2004) [37]	Anti-TNF antibody (afelimomab)	2 Parallel groups Subset by IL-6 levels < or ≥ 1,000 pg/ml	28-Day all-cause mortality
Root *et al*. (2003) [51]	Granulocyte colony stimulating factor (filgrastim)	2 Parallel groups	29-Day all-cause mortality

ACTH, adrenocorticotropic hormone; IL-1ra, IL-1 receptor antagonist; TNF, tumor necrosis factor.

**Table 2:** 28-Day all-cause mortality by study and by selected subgroups

Molecule	Study type (*n*)	Patient population	Placebo mortality (% [*n*])	Treatment mortality (% [*n*])
PAFase (Opal *et al*. 2004) [28]	Severe sepsis (1,261)	Primary	24% (150/618)	25% (161/643)
				
		APACHE II score:		
		<16	13% (16/122)	11% (16/146)
		16–20	21% (31/151)	19% (30/158)
		21–25	22% (35/156)	25% (44/173)
		>25	36% (68/188)	43% (70/162)

TFPI (Abraham *et al*. 2003) [29]	Severe sepsis (1,955)	All patients	33% (323/992)	32% (311/963)
				
		Primary:		
		INR ≥ 1.2	34% (296/874)	34% (301/880)
		INR <1.2	23% (27/118)	12% (10/83)
				
		Shock and INR ≥ 1.2:		
		Yes	35% (234/666)	36% (231/635)
		No	30% (62/208)	29% (70/245)
				
		APACHE II score and INR ≥ 1.2:		
		<20	22% (45/207)	18% (33/188)
		≥20	37% (249/665)	39% (267/689)

Low-dose steroids (Annane *et al*. 2002) [27]	Septic shock (299)	All patients	61% (91/149)	55% (82/150)
				
		Primary:		
		Nonresponder to corticotropin stimulation test	63% (73/115)	53% (60/114)
		Responder	53% (18/34)	61% (22/61)

ATIII (Warren *et al*. 2001) [35]	Severe sepsis (2,314)	Primary	39% (448/1,157)	39% (449/1,157)
				
		Shock:		
		Yes (*n *= 1,118)	43%	44%
		No (*n *= 1,191)	35%	34%
				
		SAPS II score:		
		<30% (*n *= 652)	19%	22%
		30–60% (*n *= 1,008)	41%	37%
		>60% (*n *= 654)	55%	59%

Ibuprofen (Bernard *et al*. 1997) [44]	Severe sepsis (455)	Primary	40% (92/231)	37% (83/224)
				
		Shock:		
		Yes	45% (66/147)	42% (61/146)
		No	31% (26/84)	28% (22/78)

IL-1ra (1st phase III) 1 mg/kg per hour (Fisher *et al*. 1994 [32]; Knaus *et al*. 1996 [6])	Severe sepsis (600)	Low dose	34% (102/302)	31% (91/298)
				
		Shock:		
		Yes	36% (85/239)	31% (76/244)
		No	27% (17/63)	28% (15/54)
				
		Predicted risk for death:		
		≥24%	45% (85/189)	38% (72/192)
		<24%	15% (17/113)	18% (19/106)
				
		Organ dysfunctions at baseline:		
		None	19% (22/115)	13% (14/105)
		1 or more	43% (80/187)	40% (77/193)

IL-1ra (1st phase III) 2 mg/kg per hour (Fisher *et al*. 1994) [32]	Severe sepsis (595)	High dose	34% (102/302)	29% (86/293)
				
		Shock:		
		Yes	36% (85/239)	31% (71/230)
		No	27% (17/63)	24% (15/63)
				
		Predicted risk of death		
		≥24%	45% (85/189)	35% (70/199)
		<24%	15% (17/113)	17% (16/94)
				
		Organ dysfunctions at baseline:		
		None	19% (22/115)	24% (26/110)
		1 or more	43% (80/187)	33% (60/183)

IL-1ra (2nd phase III) 2 mg/kg per hour (Opal *et al*. 1997) [34]	Severe sepsis (906)	Primary	36%	34%
				
		Evaluable	36% (126/346)	33.1% (116/350)
				
		Predicted risk for death		
		≤24% (*n *= 461)	42%	42%
		<24% (*n *= 235)	24%	18%
				
		Organ dysfunctions at baseline:		
		None	24% (22/91)	18% (17/93)
		Single	36% (47/132)	32% (43/134)
		Multiple	46% (57/123)	46% (56/123)
				
		ARDS:		
		Yes (*n *= 173)	38%	37%

E5 (1st phase III study; Greenman *et al*. 1991) [30]	Severe sepsis (468)	All Patients	41%	40%
				
		Primary:		
		Gram-negative sepsis	41% (62/152)	38% (62/164)
				
		G-ram-negative sepsis by shock status:		
		No (*n *= 137)	43%	30%
		Yes (*n *= 179)	40%	45%

E5 (2nd phase III study; Bone *et al*. 1995) [52]	Severe sepsis (530)	Primary	26% (69/266)	30% (79/264)
				
		Organ dysfunctions at baseline:		
		0 (391)	18% (36/196)	26% (51/195)
		≥1 (139)	47% (33/70)	41% (28/69)

E5 (3rd phase III study; Angus *et al*. 2000) [45]	Severe sepsis (1,090)	Primary	40% (219/544)	38% (210/546)
				
		Shock:		
		Yes	46% (145/317)	46% (140/304)
		No	33% (74/227)	29% (70/242)
				
		Hypotension:		
		Yes	43% (176/409)	43% (168/393)
		No	32% (43/135)	28% (70/242)

Lenercept (Abraham *et al*. 2001) [33]	Severe sepsis (1,342)	Primary	28% (190/680)	27% (178/662)
				
		SAPS II risk quartile:		
		0–18%	13% (23/178)	15% (25/164)
		19–31%	19% (34/178)	25% (39/155)
		32–45%	33% (53/160)	25% (43/172)
		>45%	51% (84/164)	42% (72/171)
				
		Hypotension:		
		Yes	32% (36/111)	42% (47/111)
		No	28% (159/569)	24% (132/551)
				
		Organ dysfunctions at baseline:		
		0	18% (30/164)	20% (33/164)
		1	25% (78/319)	23% (71/310)
		2	37% (54/145)	33% (44/134)
		≥3	52% (27/52)	56% (30/54)
				
		ARDS:		
		Yes	35% (35/101)	30% (31/104)

MAK 195F (Reinhart *et al*. 2001) [46]	Septic shock (446) (IL-6 level > 1,000 pg/ml)	Primary	58% (128/222)	54% (121/224)

BAYx1351 (1st phase III study) 7.5 mg/kg (Cohen and Carlet 1996) [47]	Severe sepsis (648)	Low Dose	33% (108/326)	30% (95/322)
				
		Shock:		
		Yes	46% (76/160)	38% (59/156)
		No	21% (35/166)	22% (36/166)

BAYx1351 (1st phase III study) 15 mg/kg (Cohen and Carlet 1996) [47]	Severe sepsis (649)	High dose	33% (108/326)	31% (101/323)
				
		Shock:		
		Yes	46% (76/160)	38% (61/162)
		No	21% (35/166)	25% (40/161)

BAYx1351 (2nd phase III study) 3 mg/kg (Abraham *et al*. 1995) [31]	Severe sepsis (348)	Low dose	40% (66/167)	31% (57/181)
				
		Shock:		
		Yes	43% (57/133)	37% (51/139)
		No	26% (9/34)	14% (6/42)
				
		Shock patients by APACHE II score:		
		≤24	35% (25/72)	22% (18/82)
		>24	53% (31/59)	57% (32/56)

BAYx1351 (2^nd ^phase III study) 15 mg/kg (Abraham *et al*. 1995) [31]	Severe sepsis (372)	High dose	40% (66/167)	42% (87/205)
				
		Shock:		
		Yes	43% (57/133)	45% (66/148)
		No	26% (9/34)	37%% (21/57)
				
		Shock patients by APACHE II score:		
		≤24	35% (25/72)	36% (30/84)
		>24	53% (31/59)	56% (36/64)

BAYx1351 (3rd phase III study; (Abraham *et al*. 1998) [36]	Septic shock (1,869)	Primary	43% (398/930)	40% (382/948)
				
		IL-6 concentration:		
		≤1,000 pg/ml	36% (134/369)	33% (113/341)
		>1,000 pg/ml	47% (264/561)	44% (269/607)

rhAPC (Bernard *et al*. 2001 [26]; Ely *et al*. 2003 [24])	Severe sepsis (1,960)	Primary	31% (259/840)	25% (210/850)
				
		Organ dysfunctions at baseline:		
		1	21% (43/203)	20% (42/215)
		2	26% (71/273)	21% (56/270)
		3	34% (75/218)	26% (56/214)
		4	47% (54/116)	39% (46/119)
		5	53% (16/30)	32% (10/31)
				
		IL-6 concentration quartile (low to high):		
		1st	22% (48/217)	11% (20/191)
		2nd	27% (50/189)	26% (58/220)
		3rd	33% (67/202)	29% (59/207)
		4th	44% (87/200)	31% (65/209)
				
		APACHE II score quartile:		
		3–19	12% (26/215)	15% (33/218)
		20–24	26% (57/222)	23% (49/218)
		25–29	36% (58/162)	24% (48/204)
		30–55	49% (118/241)	38% (80/210)
				
		Protrombin time:		
		<14.5 s (*n *= 103)	28%	16%
		14.5–17.4 s (*n *= 1,039)	26%	17%
		>17.4 s (*n *= 81)	51%	39%

PAFra (Dhainaut *et al*. 1998) [48]	Severe sepsis (608)	Primary	49% (153/308)	47% (140/300)

MAB-T88 (Albertson *et al*. 2003) [49]	Severe sepsis (826)	All patients	34% (141/415)	37% (152/411)
				
		Primary:		
		Enterobacteriaceae infection	31% (70/227)	34% 978/229)

NOS inhibitor (Lopez *et al*. 2004) [50]	Severe sepsis (797)	All Patients	49% (174/358)	59% (259/439)

HA-1A (Ziegler *et al*. 1991) [25]	Severe sepsis (200)	All patients	43% (118/276)	39% (100/255)
				
		Primary:		
		Gram-negative bacteremia	49% (45/92)	30% (32/105)
				
		APACHE II score:		
		≤25	38% (20/52)	20% (12/62)
		>25	60% (26/43)	48% (21/43)
				
		Shock:		
		No	40% (18/45)	27% (14/51)
		Yes	57% (27/47)	33% (18/54)

Afelimomab (Panacek *et al*. 2004) [37]	Severe sepsis (2,634)	All patients	36% (477/1,329)	32% (421/1,305)
				
		Primary:		
		IL-6 level > 1,000 pg/ml	48% (243/510)	44% (213/488)
		IL-6 level < 1,000 pg/ml	29% (234/819)	25% (208/817)

Filgrastim (Root *et al*. 2003) [51]	Pneumonia + severe sepsis (701)	All Patients	25% (90/353)	29% (101/348)

APACHE, Acute Physiology and Chronic Health Evaluation; ARDS, acute respiratory distress syndrome; ATIII, antithrombin III; IL-1ra, IL-1 receptor antagonist; INR, international normalized ratio; NOS, nitric oxide synthase; PAF, platelet activating factor; PAFra, platelet activating factor receptor antagonist; rhAPC, recombinant human activated protein C; SAPS, Simplified Acute Physiology Score; TFPI, tissue factor pathway inhibitor.

**Table 3:** Safety assessment

Study: agent	Safety assessment
Opal *et al*. (2004) [28]:	No differences between treatment groups in incidence of infectious events or serious bleeding events. No anti-PAFase antibody formation observed
Abraham *et al*. (2003) [29]: TFPI	Increased incidence of bleeding complications in TFPI treatment group (serious adverse events with bleeding 6.5% with TFPI versus 4.8% with placebo for INR ≥ 1.2; 6.0% TFPI versus 3.3% placebo for INR <1.2)
Warren *et al*. (2001) [35]: ATIII	Increased incidence of bleeding complications in ATIII treatment group (major bleeding 10.0% for ATIII versus 5.7% for placebo). No difference in event rates for other types of adverse events
Bernard *et al*. 1997 [44]: Ibuprofen	No adverse findings noted. Second episodes of sepsis occurred more often in placebo group (8.2% versus 11.1% of patients)
Fisher *et al*. (1994) [32]; Knaus *et al*. (1996) [6]: IL-1ra (1st study)	Cardiopulmonary arrest observed more often in IL-1ra treatment group (11% versus 8% of placebo patients)
Opal *et al*. (1997) [34]: IL-1ra (2nd study)	No evidence of allergic reaction. No unique clinical or laboratory adverse events were significantly more frequent in IL-1ra treatment group
Greenman *et al*. (1991) [30]: E5	Evidence of an allergic reaction noted in one study. No unique clinical or laboratory adverse events were significantly more frequent in E5 treatment group. Positive IgG anti-murine antibody response developed in 47% of E5-treated patients
Abraham *et al*. (2001) [33]: Lenercept	No unique clinical or laboratory adverse events were significantly more frequent in IL-1ra treatment group. Frequency of adverse events related to intracellular pathogen infection was similar between treatment groups
Reinhart *et al*. (2001) [46]: MAK 195F	No unique clinical or laboratory adverse events were significantly more frequent in MAK 195F treatment group. IgG human antimouse antibodies developed in 16% of MAK 195F-treated patients. No evidence of allergic reactions
Abraham *et al*. (1998) [36]: BAYx1351	Human antimouse antibody titers positive in 59.7% of patients in the BAYx1351 treatment group. The rate of bacterial superinfections or recovery from superinfections did not differ between groups. Serum sickness reported in 0.5% and 0.1% of BAYx1351-treated and placebo-treated patients, respectively
Cohen and Carlet (1996) [47]: BAYx1351	Approximately 90% of BAYx1351-treated patients developed human anti-mouse antibodies
Abraham *et al*. (1995) [31]: BAYx1351	Serum sickness reported in 2.3% and 0.0% of BAYx1351-treated and placebo-treated patients, respectively. No differences in bacterial superinfections or recovery from superinfections were noted among treatment arms. Approximately 86% of BAYx1351-treated patients developed human antimouse antibodies
Bernard *et al*. (2001) [26]: rhAPC	Increased incidence of bleeding complications in rhAPC treatment group (serious bleeding 3.5% for rhAPC versus 2.0% for placebo). No difference between treatment groups in the incidence of new infections. Neutralizing antibodies to APC not detected
Dhainaut *et al*. (1998) [48]: PAFra	No difference in the incidence of adverse events between treatment groups
Albertson *et al*. (2003) [49]: MAB-T88	Hypotension and rash noted in three MAB-T88-treated patients. Higher number of adverse events reported in MAB-T88 treatment group than in the placebo group
Lopez *et al*. (2004) [50]: NOS inhibitor	The number of patients experiencing an adverse event possibly related to study drug was higher in the 546C88 treatment group than in the placebo group (19% versus 8%). The majority of these adverse events involved the cardiovascular system (e.g. pulmonary hypertension, cardiac failure, cardiac arrest)
Panacek *et al*. (2004) [37]: afelimomab	Human antimouse antibody formation rate was 23.6% in the afelimomab group and 6.3% in the control group. No clinical sequelae were associated with antibody formation

ATIII, antithrombin III; IL-1ra, IL-1 receptor antagonist; INR, international normalized ratio; NOS, nitric oxide synthase; PAFra, platelet activating factor receptor antagonist; rhAPC, recombinant human activated protein C; TFPI, tissue factor pathway inhibitor.
